# Cost Analysis and Outcomes of Endoscopic, Minimal Access and Open Pancreatic Necrosectomy

**DOI:** 10.1097/AS9.0000000000000068

**Published:** 2021-05-07

**Authors:** Rebecca Saunders, Faye E. Hughes, Jonathan C. Evans, Howard L. Smart, Paula Ghaneh, Jayapal Ramesh, Robert Sutton, Christopher M. Halloran

**Affiliations:** From the * Department of Molecular & Clinical Cancer Medicine, University of Liverpool, UK; † Department of General & Pancreatic Surgery, Liverpool University Hospitals NHS Foundation Trust, UK; ‡ Department of Radiology, Liverpool University Hospitals NHS Foundation Trust, UK; § Department of Gastroenterology, Liverpool University Hospitals NHS Foundation Trust, UK.

## Abstract

To assess both individual patient and institutional costs as well as outcomes in patients with pancreatic necrosis who underwent either endoscopic, minimal access or open pancreatic necrosectomy. These data can be used to evaluate clinical effectiveness with a view to informing local healthcare providers.

**Background:**

Intervention for infected pancreatic necrosis is associated with a high morbidity, mortality, and long hospital stays. Minimal access surgical step-up approaches have been the gold standard of care; however, endoscopic approaches are now offered preferentially.

**Methods:**

All patients undergoing endoscopic (EN), minimal access retroperitoneal (MARPN), and open (OPN) necrosectomy at a single institution from April 2015 to March 2017 were included. Patients were selected for intervention based on morphology and position of the necrosis and on clinical factors. Patient-level costing systems were used to determine inpatient and outpatient costs.

**Results:**

Eighty-six patients were included: 38 underwent EN, 35 MARPN, and 13 OPN. Preoperative APACHEII was 6 versus 9 versus 9 (*P* = 0.017) and CRP 107 versus 204 versus 278 (*P* = 0.012), respectively. Postoperative stay was 19 days for EN versus 41 for MARPN versus 42 for OPN (*P* = 0.007). Complications occurred in 68.4%, 68.6%, and 46.2% (*P* = 0.298), whereas mortality was 10.5%, 22.9%, and 15.4% (*P* = 0.379), respectively. Mean total cost was £31,364 for EN, £52,770 for MARPN (*P* = 0.008), and £60,346 for OPN. Ward and critical care costs for EN were lower than for MARPN (ward: £9430 vs £14,033, *P* = 0.024; critical care: £5317 vs £16,648, *P* = 0.056).

**Conclusions:**

EN was at least as safe and effective as MARPN and OPN and was associated with markedly reduced hospital stay and cost, although some markers of disease severity were higher in patients undergoing MARPN and OPN. These results support EN as the preferred approach to necrosectomy, but hybrid utilization of all available techniques remains integral to optimal outcomes.

## Abstract 2

Mini Abstract

A comparison of healthcare costs and clinical outcomes was performed for 86 patients undergoing intervention for pancreatic necrosectomy. Postoperative hospital stay was reduced following endoscopic necrosectomy compared to minimal access or open necrosectomy. Total healthcare costs were reduced for patients treated by endoscopic necrosectomy.

Supplemental Digital Content is available in the text.

## INTRODUCTION

Acute pancreatitis is a potentially fatal condition that results in approximately 100 acute admissions per year for most acute hospitals in the United Kingdom.^1^ Approximately 20% of patients will develop pancreatic necrosis as a complication of acute pancreatitis with infected pancreatic necrosis subsequently occurring in 30% to 70% of this group.^2,3^ The resultant mortality is between 20% and 30%.^4–9^ In most cases of infected pancreatic necrosis, intervention is required.^10,11^

For the last decade, minimally invasive pancreatic necrosectomy has been the gold standard of management for pancreatic necrosis requiring intervention.^10,12^ More recently, endoscopic transluminal drainage and necrosectomy have been developed and shown to be an effective alternative for appropriate patients.^12,13^ Open necrosectomy is a more invasive treatment approach and, overall, its use has declined as it has been confirmed to be associated with a higher incidence of morbidity and mortality.^6,14,15^ Current evidence is inconclusive regarding the advantages of an endoscopic approach compared to minimally invasive surgical interventions in terms of clinical outcomes.^16^ The TENSION trial concluded that an endoscopic step-up approach was not superior to a surgical step-up approach^12^; however, a smaller trial found that an endoscopic approach reduced major complications and increased quality of life.^17^

Patient-level information and costing systems (PLICS) have become mandatory for acute activity in NHS hospitals from the 2018/2019 financial year, moving forward from reference costs.^18^ Costings are derived from tracking all resources used by an individual patient during their admission and calculating the actual costs incurred. This provides several advantages over the previous reference system which was based on healthcare resource group (HRG) averages and cannot be easily linked to an individual patient.^19^ HRGs are groupings of clinically similar events or treatments that are judged to use comparable levels of healthcare resource.^20^ Patient-level costing allows for more accurate comparisons between different organizations nationally and provides more accurate data for agreeing on local pricing for patient care. It is also more accessible for clinicians, allowing validation of activities and costs and a potential avenue of improving care pathways.^18^

Previous cost comparisons of endoscopic necrosectomy and a step-up surgical approach have demonstrated a trend toward reduced cost for an endoscopic approach.^12,17,21^ The aim of this study was to evaluate any potential cost benefit for a particular intervention for pancreatic necrosis by performing a clinical comparison and cost-consequence analysis using individual patient costings.

## METHODS

### Patients

All patients undergoing pancreatic necrosectomy at the Royal Liverpool University Hospital from April 1, 2015, to March 31, 2017, were included and analyzed on an intention to treat basis. Patients with admissions extending out of these times were excluded from the study. This tight time frame was chosen to accommodate whole patient episodes in which procedures were undertaken within a negotiated block contract, with a fixed budget agreed by NHS commissioners. Patients were prospectively recorded on to an electronic database.

All patients were managed in accordance with current IAP/APA guidelines.^10^ Intervention was delayed until 4 weeks post onset of acute necrotizing pancreatitis unless the clinical condition of the patient necessitated earlier drainage or laparotomy. Every patient was discussed at the weekly benign multidisciplinary team (MDT) meeting, attended by pancreatic surgeons, endoscopists, radiologists and specialist nurses. The overall management plan and the nature of any required intervention was agreed by the MDT. The mode of intervention was decided on a patient-by-patient basis, following review of the individual’s condition and the position of the necrosis. However, if the clinical condition changed, patients were treated accordingly to their emergent situation. Specific indications for intervention included clinical suspicion or documented infected necrosis, ongoing nonimproving organ failure, ongoing gastric or biliary obstruction. APACHE II scores were calculated before the initial intervention performed.

### Necrosectomy Techniques

#### Endoscopic Approach

Endoscopic (EN) transluminal drainage was the initial intervention in an endoscopic step-up approach. Under endoscopic ultrasound (EUS) guidance the optimum site for stent placement was established. Cyst puncture was performed with a 19-gauge needle and aspirated fluid was sent for culture. A lumen apposing metal stent (LAMS) with enhanced delivery system (Hot AXIOS; Boston Scientific) or biflanged metal stent (Nagi; Taewoong Medical Co. Ltd) was placed into the collection. Fluoroscopy was not routinely used for Hot AXIOS stent insertion. Necrosectomy was then performed using the “flush” method of extracavity lavage using jet pump irrigation and suction.^22^ A cap was placed on the tip of the endoscope to aid suction. Instrumentation and debridement of the cavity was avoided within the cavity. A radial expansion balloon or snares were used to unblock the stent if required. The patient underwent weekly scheduled repeat necrosectomy procedures until necrosectomy was complete; these were undertaken as an outpatient if the patient was sufficiently well. Once imaging confirmed the cavity had completely collapsed, the stent was removed, preferably within 6 weeks of insertion. Multiple metal stents or anchoring plastic stents were used at the discretion of the endoscopist.

#### Minimal Access Retroperitoneal Pancreatic Necrosectomy

Minimal access retroperitoneal pancreatic necrosectomy (MARPN) was performed as previously described.^6,7^ Initial percutaneous drainage was performed with a 12-French pigtail catheter inserted under CT guidance. In patients with central or left-sided collections, the drainage catheter was inserted via the left flank between the spleen and splenic flexure. It was possible to insert catheters anteriorly or via the right flank in patients with right-sided or complex collections. MARPN was performed under general anesthetic or sedation. The pigtail drain was exchanged for a guidewire under fluoroscopic guidance and the tract dilated up to 30 Fr using serial dilators. A sheath was inserted into the tract allowing the passage of an operating nephroscope. Necrosis was removed piecemeal under direct vision with a minimal necrosectomy on the initial procedure due to immature necrosis and to prevent bleeding. Tissue samples were sent to microbiology for culture and sensitivities. A 10- or 12-Fr nasogastric tube was sutured inside a 28-Fr chest drain and inserted into the cavity allowing postoperative irrigation. Repeat MARPNs were performed every 7–10 days until necrosectomy was complete and healthy granulation tissue was visualized. A fistulogram was performed to confirm the cavity had collapsed. The chest drain was downsized to a nasogastric tube and the patient was discharged when sufficiently fit.

#### Open Pancreatic Necrosectomy

At laparotomy, the necrotic area was exposed by transection of the gastrocolic and duodenocolic ligaments or through the space of Riolan adjacent to the ligament of Treitz, allowing blunt dissection then debridement of necrotic tissue. At least 2 wide bore drains were placed into the cavity through separate incisions and the cavity managed by closed continuous local lavage.^6,23^ Abdominal packing and second look laparotomies were not routinely performed.

For all techniques, additional percutaneous drains were inserted in to flank or loculated collections when indicated.

### Statistical Analysis

Descriptive statistics were performed on patient characteristics and outcome measures. A Chi-square test or a Kruskall–Wallis test was performed to test for statistical significance at the 5% level. Univariate logistic regression and multivariate logistic regression modeling including all factors with *P* <0.1 in univariate analysis were also performed.

### Outcomes

Length of stay including any admission in the referring center was calculated. Procedure-related adverse events (AEs) included bleeding requiring intervention, visceral perforation, problematic fistulae, and stent-related events. AEs were separated into clinical AEs: hospital acquired pneumonia, persistent sepsis, pulmonary embolism, cardiac events, and venous thrombosis and procedural AEs: bleeding, perforation, fistulae, stent migration, and stent malfunction. Additional percutaneous drainage was defined as a radiological guided drain placed into an extrapancreatic collection.

### Economic Analysis

Individual patient costs were provided by the hospital finance department for 2015–2016 and 2016–2017 financial years using PLICS. Individual patient costs for all diagnostic tests, treatment, inpatient stay, critical care stay, and outpatients were available from the Trust finance department. Endoscopy records were also interrogated to provide an accurate cost of any stents or disposable equipment used, as this is not currently represented in the PLICS data.

The drugs/treatment category included drugs, high-cost drugs, pharmacy costs, and transfusion services. Staff costs consisted of both medical staff and allied health professionals including physiotherapists, dieticians, occupational therapists and specialist nurses. The Clinical Negligence Scheme for Trusts (CNST) contributions were not included in the analysis.

A cost consequence analysis (CCA) was performed due to the difficulties in establishing one discrete outcome for the procedure required for cost-effectiveness analysis. A CCA is a practical method by which cost and outcome data can be structured to enable decision makers to improve the decision-making process.

We performed a statistical analysis of the comparative costs of EN versus MARPN versus OPN, and a subsequent cost comparison analysis of EN versus MARPN. Multiple studies have demonstrated that a minimal access approach is to be preferred over an open approach,^6,13,24^ unless there are extenuating factors necessitating an open approach; therefore, a separate analysis to compare these 2 interventions was performed to help inform our practice. Such extenuating circumstances include rapid clinical deterioration, sepsis, requiring organ support, or suspected additional intra-abdominal pathology such as visceral perforation or pancreatitis associated visceral infarction.

## RESULTS

### Clinical Outcomes

In total, 86 patients were included the analysis: 38 patients underwent EN, 35 underwent MARPN, and 13 underwent OPN. Patient demographic information is shown in Table 1. There were no differences in sex, age, etiology, number of tertiary referrals, time to intervention, or modified CT severity score between the 3 groups. There was, however, a significant difference in the maximum width of collections (113 vs 147 vs 106 mm for EN, MARPN, and OPN, respectively, *P* < 0.001) and in the location of necrosis. Of 35, 32 (91.4%) patients undergoing MARPN had necrosis in the body or tail, whereas 34 (89.5%) of 38 patients undergoing EN had necrosis in the head or body. Patients undergoing OPN and MARPN had higher APACHE II scores (6 vs 9 vs 9, *P* = 0.017) and higher CRP levels than those patients treated by EN (107 vs 204 vs 278, *P* = 0.012).

**TABLE 1.** Patient Demographics

Characteristics	Subgroup	EN (N = 38)	MARPN (N = 35)	OPN (N = 13)	Total (N = 86)	*P*
Gender, n (%)	Female	12 (31.6%)	12 (34.3%)	6 (46.2%)	30 (34.9%)	0.633
	Male	26 (68.4%)	23 (65.7%)	7 (53.8%)	56 (65.1%)	
Age, median (IQR)		58 (47, 72)	69 (49, 75)	58 (55, 71)	60 (49, 74)	0.532
Etiology of Pancreatitis, n (%)	Gallstones	23 (60.5%)	18 (51.4%)	3 (23.1%)	44 (51.2%)	0.124
	ERCP	1 (2.6%)	1 (2.9%)	2 (15.4%)	4 (4.7%)	
	Alcohol	8 (21.1%)	6 (17.1%)	5 (38.5%)	19 (22.1%)	
	Idiopathic	3 (7.9%)	1 (2.9%)	0 (0.0%)	4 (4.7%)	
	Other	2 (5.3%)	4 (11.4%)	1 (7.7%)	7 (8.1%)	
	Unknown	1 (2.6%)	5 (14.3%)	2 (15.4%)	8 (9.3%)	
Transfer from another hospital, n (%)	Yes	23 (60.5%)	26 (74.3%)	9 (69.2%)	58 (67.4%)	0.430
Days to intervention, median (IQR)		31 (11, 46)	30 (20, 45)	23 (7, 31)	30 (11, 42)	0.257
CT width (mm) of collection, median (IQR)		113 (87, 147)	147 (130, 178)	106 (75, 155)	134 (102, 160)	**<0.001**
CT severity score, n (%)	Moderate	9 (23.7%)	8 (22.9%)	2 (15.4%)	19 (22.1%)	0.882
	Severe	29 (76.3%)	27 (77.1%)	11 (84.6%)	67 (77.9%)	
Day 7 post admission RLUH CRP, median (IQR)		107 (55, 228)	204 (107, 244)	278 (183, 335)	183 (93, 248)	**0.012**
Preoperative ITU stay, n (%)	Yes	5 (13.2%)	9 (25.7%)	6 (46.2%)	20 (23.3%)	**0.047**
Site, n (%)	Head	10 (26.3%)	3 (8.6%)	3 (23.1%)	16 (18.6%)	**0.028**
	Body	24 (63.2%)	20 (57.1%)	5 (38.5%)	49 (57.0%)	
	Tail	4 (10.5%)	12 (34.3%)	5 (38.5%)	21 (24.4%)	
Total APACHE II score, median (IQR)		6 (2, 9)	9 (5, 12)	9 (6, 16)	7 (4, 11)	**0.017**

Significant results are indicated in bold.

Postoperative outcomes are shown in Table 2. The median (IQR) total length of stay was significantly different: 52 (29, 74) days for EN patients, 74 (55, 102) days for MARPN, and 63 (53, 79) days for OPN (*P* = 0.007). The postoperative length of stay was lower in the EN group compared to MARPN and OPN (19 vs 41 vs 42 days, *P* < 0.001). In-patient mortality was 4 (10.5%) for EN, 8 (22.9%) for MARPN, and 2 (15.4%) for OPN (*P* = 0.379). Overall AEs occurred in 26 (68.4%) patients undergoing EN, 24 (68.6%) for MARPN, and 6 (46.2%) for OPN. Procedural-related AEs were higher in the EN group (*P* = 0.002), whereas clinical AEs were higher in the MARPN group (*P* = 0.046). Confirmed infected necrosis was significantly higher for MARPN and OPN [32 (91.4%) and 11 (84.6%) versus 14 (36.8%) for the EN group], *P* < 0.001, but only 14 patients undergoing EN had samples sent for culture, all of whom had positive cultures. The common organisms found on culture were *Escherichia coli*, *Enteroccocus* species, *Klebsiella* species, and *Candida albicans*. There was no difference in difference in microbiota cultured between groups. There was no significant difference in the number of patients requiring additional percutaneous drainage (*P* = 0.115). The median (IQR) number of necrosectomies were 4 (2, 5) for EN, 2 (1, 3) for MARPN, and 1 for OPN (*P* < 0.001).

**TABLE 2.** Postoperative Descriptive Statistics

Outcomes	EN (N = 38)	MARPN (N = 35)	OPN (N = 13)	Total (N = 86)	*P*
Total length of stay (d), median (IQR)	52 (29, 74)	74 (55, 102)	63 (53, 79)	63 (45, 85)	**0.007**
Length of stay in RLBUHT (d), median (IQR)	28 (17, 50)	55 (39, 81)	48 (36, 58)	42 (26, 64)	**<0.001**
Postoperative length of stay (d), median (IQR)	19 (8, 41)	41 (28, 70)	42 (26, 54)	34 (19, 55)	**<0.001**
In-patient mortality, n (%)	4 (10.5%)	8 (22.9%)	2 (15.4%)	14 (16.3%)	0.379
90-d mortality, n (%)	4 (10.5%)	8 (22.9%)	3 (23.1%)	15 (17.4%)	0.323
AEs, n (%)	26 (68.4%)	24 (68.6%)	6 (46.2%)	56 (65.1%)	0.298
AE (procedure), n (%)	19 (50.0%)	5 (14.3%)	2 (15.4%)	26 (30.2%)	**0.002**
AE (clinical), n (%)	14 (36.8%)	23 (65.7%)	6 (46.2%)	43 (50.0%)	**0.046**
Infected necrosis, n (%)	14 (36.8%)	32 (91.4%)	11 (84.6%)	57 (66.3%)	**<0.001**
Total ITU stay (d), median (IQR)	0 (0, 0)	0 (0, 5)	3 (0, 22)	0 (0, 3)	**0.003**
Percutaneous drainage, n (%)	9 (23.7%)	16 (45.7%)	4 (30.8%)	29 (33.7%)	0.115
No. necrosectomies, median (IQR)	4 (2, 5)	2 (1, 3)	1 (1, 1)	2 (1, 4)	**<0.001**

Significant results are indicated in bold.

Table 3 shows specific complications occurring in individual groups. There was no significant difference in complications between the interventions. The incidence of persistent pancreatic fistulae was lower after EN compared to MARPN or OPN; however, this did not reach statistical significance (*P* = 0.104). In the endoscopic group, stent-related problems occurred in 16 (42.1%) patients.

**TABLE 3.** Adverse Events

Characteristics	EN (N = 38)	MARPN (N = 35)	OPN (N = 13)	Total (N = 86)	*P*
Bleeding, n (%)	1 (2.6%)	4 (11.4%)	1 (7.7%)	6 (7.0%)	0.304
Fistula, n (%)	1 (2.6%)	5 (14.3%)	2 (15.4%)	8 (9.3%)	0.104
HAP, n (%)	2 (5.3%)	5 (14.3%)	1 (7.7%)	8 (9.3%)	0.420
Cardiac, n (%)	2 (5.3%)	2 (5.7%)	0 (0.0%)	4 (4.7%)	1.000
Persistent sepsis, n (%)	3 (7.9%)	7 (20.0%)	2 (15.4%)	12 (14.0%)	0.339
PE, n (%)	1 (2.6%)	1 (2.9%)	0 (0.0%)	2 (2.3%)	1.000
PV thrombosis, n (%)	2 (5.3%)	0 (0.0%)	0 (0.0%)	2 (2.3%)	0.636
SMV thrombosis, n (%)	6 (15.8%)	2 (5.7%)	1 (7.7%)	9 (10.5%)	0.404
Readmission, n (%)	10 (26.3%)	11 (31.4%)	3 (23.1%)	24 (27.9%)	0.850
Perforation, n (%)	2 (5.3%)	1 (2.9%)	0 (0.0%)	3 (3.5%)	1.000

Univariate logistic regression analysis (see Supplemental Table 1, http://links.lww.com/AOSO/A38) was performed for mortality. This demonstrated that the factors associated with increased mortality in the whole cohort of patients were age [odds ratio (OR) 1.042, 95% confidence interval (CI) 1.001–1.086] transfer from another center (OR 9.419, 95% CI 1.176–75.441), APACHEII score (OR 1.13, 95% CI 1.048–1.230), preoperative ICU stay (OR 6.896, 95% CI 2.121–22.419), and percutaneous drainage (OR 4.386, 95% CI 1.400–13.736). Multivariate logistic regression models (see Supplemental Table 2, http://links.lww.com/AOSO/A39) were performed for the outcome of AEs. They were not performed for mortality due to the small number of events. Patients with pancreatitis secondary to alcohol (OR 0.191, 95% CI 0.046–0.799) were less likely to suffer AEs than those with an etiology of gallstones. A longer length ICU stay was also associated with increased AEs (OR 1.112, 95% CI 1.008–1.227).

### Economic Outcomes

**FIGURE 1.**
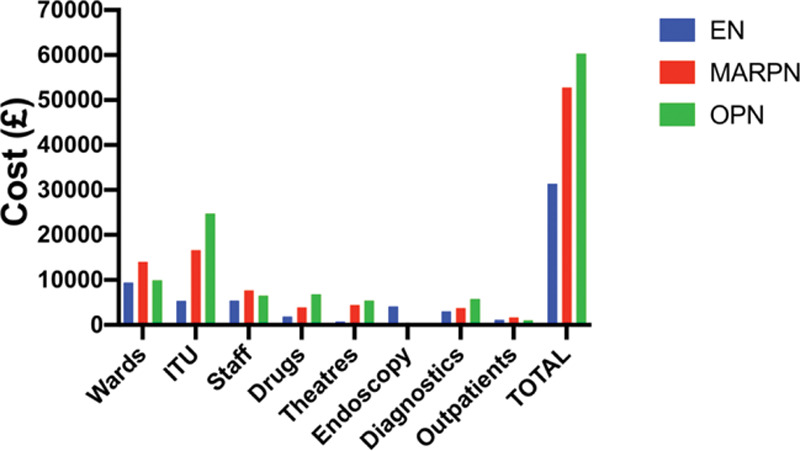
Graph showing average cost (£) per patient for EN, OPN, and MARPN.

Individual patient costs were calculated using PLICS are summarized in Table 4 and Figure 1. The mean overall cost per patient was £30,981 for patients treated by EN, £52,357 for MARPN, and £60,077 for OPN (*P* = 0.006). Similarly, the ward and intensive care costs were £9430 and £14,033; £9890 (*P* = 0.089) and £5317; £16,648 and £24,722 for EN, MARPN, and OPN, respectively (*P* = 0.001).

**TABLE 4.** Summary Table of the Average Cost (£) per Patient for EN, OPN, and MARPN

Department	EN (£)	MARPN (£)	OPN (£)	*P*
Wards	9430	14,033	9890	0.089
ITU/critical care	5317	16,648	24,722	**0.001**
Staff	5358	7648	6501	0.298
Drugs/treatment	1852	3910	6807	**0.024**
Theaters	784	4420	5369	**0.001**
Endoscopy	4135	245	0	**0.001**
Diagnostic tests	2970	3762	5738	0.378
outpatients	1135	1691	1050	0.611
TOTAL	30,981	52,357	60,077	**0.006**

Significant results are indicated in bold.

HAP, hospital acquired pneumonia; PE, pulmonary embolus; PV, portal vein; SMV, superior mesenteric vein.

**FIGURE 2.**
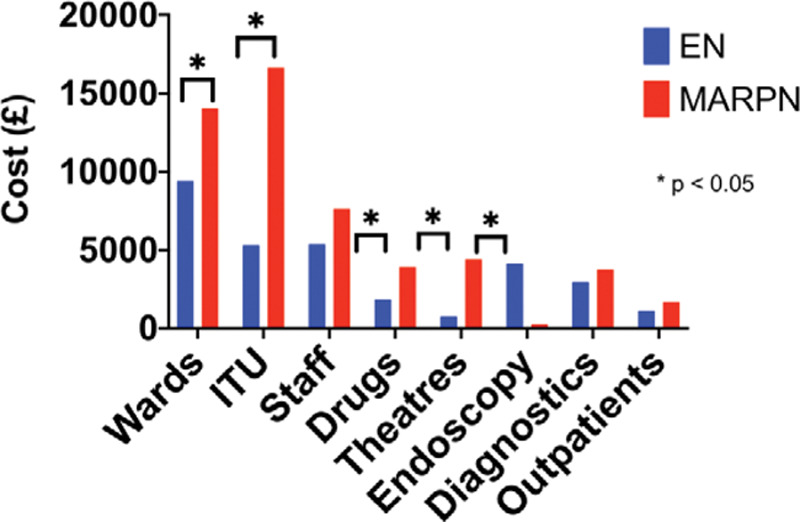
Comparison of average costs for EN vs MARPN (£).

Table 5 and Figure 2 show a cost comparison of EN and MARPN which demonstrates a significantly lower average total cost for EN (£30,981) when compared to MARPN (£52,537) (*P* = 0.004). The cost of ward care (£9430 vs £14,033, *P* = 0.035) and medication (£1852 vs £3910, *P* = 0.006) were also significantly lower for patients undergoing EN. The operating room costs in the MARPN group were comparable with endoscopy costs for patients managed by EN (£4420 and £4135).

**TABLE 5.** Cost Comparison for EN vs MARPN (£)

Average Costs per Patient	EN (£)	MARPN (£)	*P*
Wards	9430	14,033	**0.035**
ITU	5317	16,648	**0.025**
Staff	5358	7648	0.168
Medication/ treatment	1852	3910	**0.006**
Theaters/OR	784	4420	**0.001**
Endoscopy	4135	245	**0.001**
Diagnostic tests	2970	3762	0.148
Outpatients	1135	1691	0.551
Total	30,981	52,537	**0.004**

Significant results are indicated in bold.

## DISCUSSION

This study has investigated the actual cost alongside clinical outcomes for different approaches for the management of pancreatic necrosis in a real-world setting. All patients undergoing EN, MARPN, or OPN at a tertiary center over 2 financial years, within a block UK NHS financial contract period, were included in the analysis. The results give an accurate representation of current costs for treating this complex cohort of patients with long and resource heavy inpatient stays. The most important finding is that patients undergoing EN had outcomes equivalent to those undergoing MARPN or OPN, with reduced inpatient stays and reduced treatment costs. Despite the less severe disease profile of patients undergoing EN; however, the average cost of EN was £30,981, whereas the NHS National Tariff for pancreatic necrosectomy for the 2019/2020 financial year was only £21,212, substantially less than it costs a center to treat the majority of these patients.^25,26^ Those responsible for commissioning and allocating resources for health services should ensure that these essential costs are met.

The average cost for managing a patient with MARPN was over £20,000 more expensive than for EN. The increased cost is largely due to the significantly longer length of stay of the MARPN patients on both a surgical ward and ICU. The increased length of stay for MARPN of approximately 3 weeks is likely to be related to many factors, including being performed in a sicker cohort of patients and the need to prolong hospital stay until drain irrigation has been discontinued and drain downsized. Contrastingly, our protocol for EN is an initial transgastric drainage, followed by flush necrosectomy at 7 days; clinically well and suitable patients can then be discharged, without the need for irrigation, with EN performed on a weekly outpatient basis until the necrotic collection has resolved. It is reassuring that there was no increase in readmission following EN, suggesting that the protocol is safe. The finding of a reduced length of stay for endoscopic necrosectomy is consistent with previously published studies.^12,17^

OPN was associated with higher costs than the less invasive approaches, as OPN was associated with higher ICU costs and longer ICU stays. Previously published studies have found increased morbidity with OPN compared to minimal access techniques.^6,7,13,24,27^ Bakker et al^13^ observed a trend toward increased ITU stays for OPN, but this did not reach statistical significance; they also reported an increased inflammatory response following open surgical necrosectomy compared to EN. This may be partly responsible for the increased ITU stay and costs found for OPN. However, the OPN cohort in our analysis may have been more physiologically unstable initially, as we report higher CRP values and APACHEII scores for the OPN compared to the EN patients.

This study is an observational analysis with intervention decided by the MDT rather than by randomization. We started performing EN shortly before the time frame included in this study, so the learning curve period for the technique is included in these data. As clinicians became more experienced with the technique, it was performed on a wider range of patients, including those on ICU and those with less favorable collections. The preoperative patient characteristics show that MARPN and OPN were performed in patients with higher APACHEII scores, higher CRP and associated with more ICU admissions then EN, limiting direct comparisons. Any patient who deteriorated was reassessed and the plan of intervention adjusted accordingly. Patients waiting for EN (who required a specialist endoscopist) or patients waiting for MARPN (who required an interventional radiology guidewire/drain placement as part of the procedure), in whom appropriate infra-structure was not immediately available and in whom it was felt life was in danger, underwent surgical intervention. Judgments of best care are commonplace in tertiary units, dealing with inter-regional transfers at high volume.

The site of the pancreatic necrosis has implications for the approach chosen; for EN, the collection has to be accessible via the transgastric or transduodenal route, whereas collections in the tail may be inaccessible. For MARPN, collections have to be approached via the flanks, although central or right-sided collections may also be accessible percutaneously in some patients.

Treatment algorithms have not been widely used for pancreatic necrosis, given the heterogeneity of the disease and variations in local expertise. One group is continuing to develop an algorithm to define the role of surgical approaches by time from onset of pancreatitis and hemodynamic status.^28^ We feel the optimal way to approach pancreatic necrosis is to use a treatment algorithm taking into account the location of the necrosis and physiological condition of the patient to determine the management approach. This includes percutaneous drains, endoscopic, minimal access, and complex minimal access including single-port necrosectomy,^29^ open necrosectomy or a combination of the above. Our work is ongoing.

## ACKNOWLEDGMENTS

PLICS data were provided by Ann-Marie Colligan, Royal Liverpool University Hospital NHS Trust. Alan Haycox (University of Liverpool) provided expertise and advice on health economics. Rebecca Griffin (University of Liverpool) provided statistical support.

## Supplementary Material
