# Supplementary material for: Cost Analysis and Outcomes of Endoscopic, Minimal Access and Open Pancreatic Necrosectomy
Source: Ann Surg Open. 2021 May 7;2(2):e068. doi: 10.1097/AS9.0000000000000068 (PMC7610877; doi:10.1097/AS9.0000000000000068)
Supplement: Supplementary file 1 [file as9-2-e068-s001.pdf]

Characteristics	Sub-group	Odds Ratio	95% Confidence Interval	P-value
**Intervention**	OPN	5.185	(0.979, 27.450)	0.053
	MARPN	4.038	(0.994, 16.405)	0.051
**Sex**	Male	1.222	(0.381, 3.917)	0.736
**Age**		1.042	(1.001, 1.086)	**0.046**
**Cause of Pancreatitis**	ERCP	7.800	(0.891, 68.304)	0.064
	Alcohol	2.080	(0.491, 8.808)	0.320
	Idiopathic	0.000	(0.000, ∞)	0.994
	Other	5.850	(1.004, 34.100)	0.050
	Unknown	2.600	(0.408, 16.559)	0.312
**Transfer from another hospital**	Yes	9.419	(1.176, 75.441)	**0.035**
**Days to intervention**		1.005	(0.985, 1.024)	0.653
**CT width (mm)**		1.008	(0.995, 1.021)	0.217
**CT severity**	Severe	5.192	(0.640, 42.145)	0.123
**Day 7 CRP**		1.005	(1.000, 1.010)	0.059
**Preoperative ICU stay**		6.896	(2.121, 22.419)	**0.001**
**Site**	Body	1.575	(0.303, 8.190)	0.589
	Tail	2.187	(0.365, 13.100)	0.391
**Total length of stay (days)**		1.008	(0.996, 1.020)	0.179
**Length of stay in RLBUHT (days)**		1.010	(0.993, 1.027)	0.244
**Postoperative length of stay (days)**		1.012	(0.995, 1.030)	0.171
**Adverse event**	Yes	4.667	(0.984, 22.138)	0.052
**Infected Necrosis**	Yes	4.395	(0.926, 20.871)	0.062
**Length of stay in ICU (days)**		1.092	(1.038, 1.148)	**0.001**
**Total APACHE II score**		1.135	(1.048, 1.230)	**0.002**
**Percutaneous drainage**	Yes	4.386	(1.400, 13.736)	**0.011**
**Number of Necrosectomy**		0.972	(0.750, 1.258)	0.828
