# Supplementary material for: Cost Analysis and Outcomes of Endoscopic, Minimal Access and Open Pancreatic Necrosectomy
Source: Ann Surg Open. 2021 May 7;2(2):e068. doi: 10.1097/AS9.0000000000000068 (PMC7610877; doi:10.1097/AS9.0000000000000068)
Supplement: Supplementary file 2 [file as9-2-e068-s002.pdf]

Characteristics	Sub-group	Odds Ratio	95% Confidence Interval	P-value
**Cause of Pancreatitis**	ERCP	0.067	(0.004, 1.096)	0.058
	Alcohol	0.191	(0.046, 0.799)	**0.023**
	Idiopathic	2.014	(0.177, 22.848)	0.572
	Other	0.118	(0.011, 1.299)	0.081
	Unknown	2.978	(0.293, 30.309)	0.357
**Day 7 CRP**		1.005	(0.999, 1.011)	0.078
**Length of stay in ICU (days)**		1.112	(1.008, 1.227)	**0.034**
